# Prevention of veno-occlusive disease/sinusoidal obstruction syndrome: a never-ending story and no easy answer

**DOI:** 10.1038/s41409-023-02007-2

**Published:** 2023-05-25

**Authors:** Selim Corbacioglu, Stephan A. Grupp, Paul Gerard Richardson, Rafael Duarte, Antonio Pagliuca, Tapani Ruutu, Kris Mahadeo, Enric Carreras

**Affiliations:** 1 University of Regensburg, Regensburg, Germany; 2 The Children’s Hospital of Philadelphia, Philadelphia, PA USA; 3 Jerome Lipper Multiple Myeloma Center, Dana-Farber Cancer Institute, Harvard Medical School, Boston, MA USA; 4 Department of Hematology, Hospital Universitario Puerta de Hierro Majadahonda, Madrid, Spain; 5 King’s College Hospital and Anthony Nolan Research Institute, London, UK; 6 Comprehensive Cancer Center, Helsinki University Hospital, Helsinki, Finland; 7 Duke University Children’s Hospital, Durham, NC USA; 8 Josep Carreras Foundation & Leukemia Research Institute, (Hospital Clínic/Barcelona University Campus), Barcelona, Spain

**Keywords:** Phase III trials, Stem-cell research, Randomized controlled trials

The Harmony Trial (NCT02851407), a prospective randomized trial conducted among adults and children, reported that defibrotide was not effective for the prevention of veno-occlusive disease/sinusoidal obstruction syndrome (VOD/SOS) [1]. Importantly no safety concerns were raised [2], but consecutively, in May 2022, the European Medicines Agency (EMA) recommended not to use defibrotide for prophylaxis of VOD/SOS (https://www.ema.europa.eu/en/medicines/dhpc/defitelio-defibrotide-do-not-use-prophylaxis-veno-occlusive-disease-vod-after-post-hematopoietic).

A decade ago, another prospective randomized trial, the Pediatric Prevention Trial (NCT00272948), clearly demonstrated efficacy in the prevention of VOD/SOS [3]. Prospective randomized trials are considered the so-called “gold standard” of clinical medicine. What happened that such two prospective randomized trials yielded apparently contradictory results?

The Pediatric Prevention Trial was conceptualized between 2001 and 2005 and recruited 360 pediatric patients considered at high risk for VOD/SOS between 2006 and 2009, being informed by prior and ongoing treatment studies at the time in which defibrotide had shown consistent efficacy in the setting of established disease [4]. The sample size calculation generated back in 2005 was based on the presumed incidence in high-risk pediatric patients of up to 30%. In order to reduce this incidence by half, to 15%, the sample size calculation resulted in accrual of 270 patients for both arms. In 2008, the interim analysis revealed that the actual incidence of VOD/SOS in this high-risk population based on investigator assessment was only 20% according to the modified Seattle criteria. Consequently, the sample size was adjusted as part of a planned interim analysis to 360 patients. Eventually the trial demonstrated efficacy in the competing risk analysis of the intent-to-treat population reducing the incidence from 20 to 12% (*p* = 0.049; per protocol population: *p* = 0.022) [3].

Whereas the Pediatric Prevention Trial focused exclusively on the highest risk pediatric population, the Harmony Trial enrolled pediatric and adult patients and enrolled patients where high risk among other criteria was defined as a myeloablative conditioning (MAC) with more than two (not further defined, see below) alkylators or TBI plus one or more alkylators. Current high-risk populations in adult disease—such as those exposed to sirolimus—were excluded, due to regulatory considerations on the part of the sponsor at the time. Almost half of the patients enrolled were adults and half of the diagnoses were acute leukemias. Only 15% and 7% of the enrolled patients, respectively, were very high-risk pediatric patients with neuroblastoma and osteopetrosis.

In 2010, a meta-analysis including 25,000 pediatric and adult patients revealed an average VOD/SOS incidence of 13.7% [5]. The reported incidence in children was around 20% [6, 7] which was later confirmed in the Pediatric Prevention Trial with an overall incidence of 22.2% and an even higher incidence of 29.3% reported in infants [3].

In 2018, a study including 13,000 patients defined the incidence in the highest risk population as ~18% with the most significant non-linear relationship existing between age and risk of VOD/SOS [8], and a contemporaneous meta-analysis including more than 27,000 patients confirmed a VOD/SOS incidence of 15% [9, 10]. In summary, an average VOD/SOS incidence of ~15% can be considered realistic with a persistent and consistently higher incidence of VOD/SOS in children, particularly in infants as compared to adults.

The Harmony Trial had an adaptive design with a calculated sample size of 400 patients based on a presumed incidence of 28% in the best supportive care (BSC) group. This incidence in the control arm was not even reached in the 10 years older trial in high-risk pediatric patients as described above. With a decade between the two trials with scientific advancements that improved the safety of hematopoietic stem cell transplantation (HSCT) significantly, the presumed incidence was not realistic. One example, treosulfan, albeit an alkylator such as busulfan, demonstrates a reduced endothelial toxicity profile. This is most evident in infantile malignant osteopetrosis (IMO) with a historical incidence of VOD/SOS as high as 60% [3]. Shadur et al. [11] reported no VOD/SOS on 31 IMO patients using a treosulfan, fludarabine and thiotepa-based MAC regimen with an overall survival of 100%.

In retrospect, these observations suggest that a power calculation based on an overestimated incidence of VOD/SOS of 28%, in an adult/pediatric study, resulting in an estimated sample size of 400, was markedly insufficient to detect a significant reduction in a non-composite endpoint, i.e., the incidence of VOD/SOS.

Moreover, this was not the primary endpoint ultimately chosen by the sponsor, as based on guidance from regulatory authorities. The primary endpoint of the Harmony Trial was required to be VOD/SOS-free survival by day 30 post-HSCT. There proved to be even greater challenges with this composite endpoint than simply an overestimate of the incidence. VOD/SOS is graded according to severity and only the severe/very severe grades are associated with a mortality of up to 80% [5] whereas the mortality of mild/moderate cases is by comparison very low. VOD/SOS with multiorgan failure (MOF; sometimes referred to as multiorgan dysfunction) occurs in an estimated 30–40% of patients with VOD/SOS after HSCT [12]. Therefore, considering a population of 40% out of 15% that are at risk of treatment-related mortality due to VOD/SOS, the trial population at risk for this composite endpoint was further reduced to less than 7%. Szmit et al. analyzed the impact of preemptive treatment with defibrotide by comparing in a single institution Seattle criteria with the pediatric EBMT criteria [13]. They not only found a significantly improved survival but also a shortened median hospitalization by 12 days correlating with a reduced admission to the pediatric intensive care unit (PICU) (3.9% vs. 14.6%) (personal communication), clearly reflecting morbidity from VOD as a significant endpoint for treatment efficacy. In conclusion, survival is a crude primary endpoint in defining the benefit of an intervention in VOD/SOS by ignoring the substantial morbidity related to this disease.

In addition, and importantly, a statistically meaningful difference in outcome for the endpoint of survival was diluted in both trials by the required and necessary equipoise of allowing defibrotide use for the treatment of any emergent VOD/SOS in the BSC group.

**Fig. 1:**
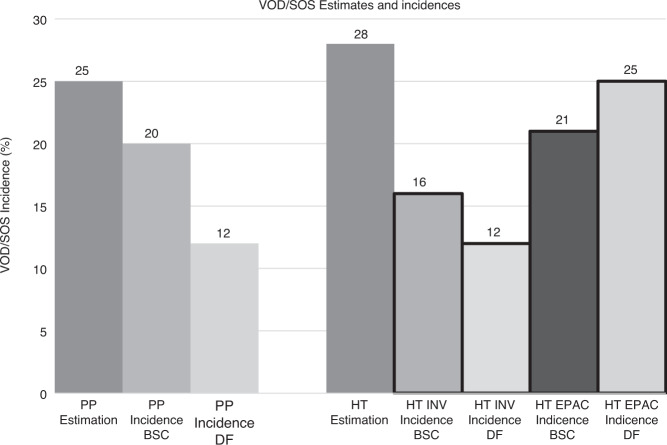
VOD/SOS estimates and incidences in the Pediatric Prevention trial (PP) and the Harmony Trial (HT). PP Pediatric Prevention Trial, BSC best supportive care, HT Harmony trial, HT INV incidences of the Harmony trial according to the investigators, HT EPAC incidences of the Harmony trial according to the EPAC.

In designing the study, there were also concerns about correctly and reproducibly diagnosing VOD/SOS. To address this, the Harmony Trial implemented an independent Endpoint Adjudication Committee (EPAC) to provide centralized assessment of the primary endpoint (VOD/SOS-free survival by day 30 post-HSCT) which comprised 4 independent HSCT specialists. Unexpectedly, the EPAC assessment of VOD/SOS resulted in detection of significantly more cases of VOD/SOS (25% for prophylaxis vs. 21% for BSC) than that recorded by the investigators at the bedside (12% for prophylaxis vs. 16% for BSC) (Fig. 1). Disagreements between EPAC and investigators proved asymmetric: there were many more cases of disagreement between EPAC and investigator in the direction of the EPAC calling VOD/SOS when the investigator did not, compared to the opposite scenario. The primary endpoint was based on EPAC assessment alone. This significant discrepancy was not factored into the decision by the data and safety monitoring committee (DSMC) to stop the trial for futility at the time of interim analysis, which was based upon an apparent lack of efficacy in a limited sample size, a decision worsened by its timing during the pandemic and legitimate concerns regarding the pace of future enrollment. It is not possible to know if a trial with a more realistic sample size; recruited over a longer period; using real world bedside assessment of VOD/SOS, and designed to assess the impact of defibrotide prophylaxis based upon VOD/SOS incidence, might have reached a different conclusion, and above all, if it was focused on current higher risk patients, a conclusion that might then have matched the outcome of the previous Pediatric Prevention Trial [3].

Given the totality of evidence, and in particular when faced with pediatric patients at very high risk of VOD/SOS, we would point to the results of the Pediatric Prevention Trial as providing justification for use of prophylactic defibrotide in this smaller and highly selected group of patients for which there is exquisite unmet medical need. Given the substantive methodological concerns raised with the Harmony Trial, we still regard this as an open question, and would not use this latter result over others to recommend against the use of defibrotide as prophylaxis for patients at high risk. Therefore, before drawing any final conclusions, we advocate the consideration of the entire body of clinical benefit seen to date, especially when there is such a discrepancy in outcome in two prospective randomized trials and would recommend further carefully designed studies in high-risk groups. The consequences of not recommending any prevention of VOD/SOS are already devastating for a substantial number of transplant patients, and especially for high-risk infants, being amongst the most vulnerable populations undergoing HSCT, as well as others.
